# The Gene Set Builder: collation, curation, and distribution of sets of genes

**DOI:** 10.1186/1471-2105-6-305

**Published:** 2005-12-21

**Authors:** Dimas Yusuf, Jonathan S Lim, Wyeth W Wasserman

**Affiliations:** 1 Centre for Molecular Medicine and Therapeutics (CMMT), Child & Family Research Institute, Vancouver, Canada; 2 Department of Medical Genetics, University of British Columbia, Vancouver, Canada

## Abstract

**Background:**

In bioinformatics and genomics, there are many applications designed to investigate the common properties for a set of genes. Often, these multi-gene analysis tools attempt to reveal sequential, functional, and expressional ties. However, while tremendous effort has been invested in developing tools that can analyze a set of genes, minimal effort has been invested in developing tools that can help researchers compile, store, and annotate gene sets in the first place. As a result, the process of making or accessing a set often involves tedious and time consuming steps such as finding identifiers for each individual gene. These steps are often repeated extensively to shift from one identifier type to another; or to recreate a published set. In this paper, we present a simple online tool which – with the help of the gene catalogs Ensembl and GeneLynx – can help researchers build and annotate sets of genes quickly and easily.

**Description:**

The Gene Set Builder is a database-driven, web-based tool designed to help researchers compile, store, export, and share sets of genes. This application supports the 17 eukaryotic genomes found in version 32 of the Ensembl database, which includes species from yeast to human. User-created information such as sets and customized annotations are stored to facilitate easy access. Gene sets stored in the system can be "exported" in a variety of output formats – as lists of identifiers, in tables, or as sequences. In addition, gene sets can be "shared" with specific users to facilitate collaborations or fully released to provide access to published results. The application also features a Perl API (Application Programming Interface) for direct connectivity to custom analysis tools. A downloadable Quick Reference guide and an online tutorial are available to help new users learn its functionalities.

**Conclusion:**

The Gene Set Builder is an Ensembl-facilitated online tool designed to help researchers compile and manage sets of genes in a user-friendly environment. The application can be accessed via .

## Background

Grouping genes into "sets" has become an intuitive and commonplace practice in bioinformatics and genomics research. Many bioinformatics applications can analyze sequential, structural, functional, and expressional ties between genes in a given set. For instance, the oPOSSUM system can identify over-represented transcription-factor binding sites in a group of co-expressed genes [1]. Similarly, GOToolBox can identify Gene Ontology terms which are over-represented in the annotations of a set of genes [2]. In short, a new generation of analysis methods requires – as inputs – sets of genes.

Despite an abundance of these multi-gene investigative tools, to our knowledge, no published tools exist which help researchers compile, store, and share sets of genes. Consequently, researchers often revert to the time-tested method of copying and pasting gene identifiers and annotations into a spreadsheet or a text file. While this technique may be convenient for building small sets of genes, it becomes burdensome for large or shared collections.

In this paper, we present the Gene Set Builder, a web-based system designed to help researchers quickly build, sort, and annotate sets of genes in a user-friendly environment. This application features a "point and click" interface that lets users search and import genes in batches; synchronize missing and outdated gene annotations with currently available information; compile and export gene sets as FASTA sequences, cDNA transcripts, tables, or as lists of identifiers; share data with other users; and create sets of homologs to facilitate comparative studies across species.

## Construction and content

### Code

Gene Set Builder is written in the Perl programming language. The Perl backend uses several third-party modules including CGI, DBI, DBD-mysql, and the GeneLynx API [3]. Components of BioPerl [4] are used to access genomic and cDNA sequences. Similar to other web-based applications, the Perl scripts are executed through a CGI to generate a HTML-based user interface.

### Database

User-created information is maintained in a password-protected MySQL database. An outline of the database structure is shown in Additional file 1.

### User interface

**Figure 1:**
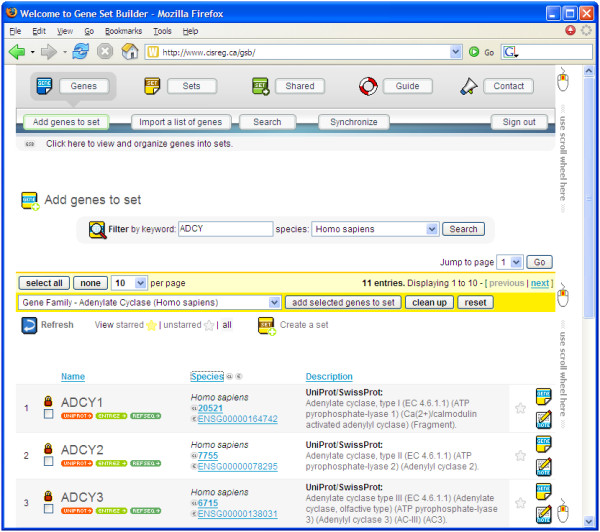
**A screen capture of Gene Set Builder. **This "special edition" user interface utilizes a Flash-based navigation system, complete with animation and tool tips.

Driven by HTML, JavaScript, and Macromedia Flash, Gene Set Builder's interface is designed to be intuitive, flexible, and graphically-rich. It features a navigation system with three main categories: "Genes", "Sets", and "Shared" (Figure 1). When users click on a category, a list of relevant functions is displayed. For instance, clicking on the "Genes" category will display the "Add genes to set", "Import a list of genes", "Search", and "Synchronize" functions. An HTML-based navigation system ("classic interface") is available to accommodate web browsers without the Flash plug-in.

### API

A Perl Application Programming Interface has been developed to help advanced users retrieve data directly from the Gene Set Builder database. This API can obtain gene and set annotations including names, symbols, descriptions, comments, confidence ratings, and identifiers.

## Utility

Here we discuss the use of Gene Set Builder: building and sharing gene sets, data annotation, exporting, and using the API.

### Tutorial

We have created a number of resources to help new users learn how to use Gene Set Builder. On the homepage, one can access multimedia walkthroughs of the system's essential features, and download a Quick Reference guide.

### Building and sharing a set of genes

Genes can be imported to the "Add genes to set" staging area in two ways: (1) users can search for genes individually via search engines which accesses BioMART [5] and GeneLynx, or (2) users can input gene or protein identifiers from diverse resources. For convenience, this mass import tool accepts gene symbols (e.g. HUGO-approved symbols [6]), Affymetrix gene identifiers or accession numbers from Entrez Gene [7], Ensembl, UniProt [8], Swiss-Prot [9] or RefSeq [10]. From the genes returned to the staging area, users can select specific genes for inclusion in new or existing sets.

Sets can be created from other sets as well. The "Create a homolog set" tool can be used to generate a set of homologous genes from an existing single or multi-species set. This feature is based on Ensembl homology annotations. In addition, users can make copies of sets.

The attributes and contents of a gene set can be modified: users can add or remove genes from sets at any time; sets can be commented, shared, "unshared", renamed, deleted, rendered accessible via the API, or protected from accidental changes via a "lock" feature. Users can also make their gene sets available for public access via the "share set" feature. While visitors can view, export, and copy a shared set, only the set's owner has the privilege to change, delete, or withdraw the set from the public domain. For convenience and testing purposes, Gene Set Builder is preloaded with widely-used sets, such as the ESR Dataset [11] and the *S. cerevisiae *Cell Cycle-regulated Gene Set [12].

### Custom annotations and data management

Gene Set Builder can retrieve UniProt, Entrez Gene, RefSeq, and GeneLynx identifiers via BioMART annotations. This task is mediated by the "Synchronize" feature, which can be found under "Genes" in the menu. Users may annotate the confidence of a gene's membership in a set via a 5-point scale displayed as a column of star icons. Users can also attach comments to genes in a general and set-specific context. In addition to comments and confidence ratings, we have included search functions to locate or eliminate genes in the workspace by species or keyword. This search engine supports Boolean syntaxes such as AND, OR, and NOT. Users can tag genes and sets so they can be easily retrieved in the future.

### Exporting

To accommodate analysis tools which accept gene identifiers as inputs such as oPOSSUM and GoMiner, a set of genes can be exported as a list of Entrez Gene, Ensembl, RefSeq, UniProt, or GeneLynx identifiers. When exporting as a list, the user can exclude genes based on their confidence ratings and/or species. FASTA-formatted sequences can also be created, with the option to specify upstream and downstream flanking basepairs for regulatory sequence analysis. Gene Set Builder can also generate a table populated with gene identifiers and descriptions, which the user can save and open with a word processing or spreadsheet application.

### Using the API

The API-enabling feature in Gene Set Builder is treated as an Export function which copies the desired gene set into the "open-access" portion of the database. Data stored in this area can be retrieved via the Perl API or a MySQL client. When exporting sets in this format, users can choose to divulge only specific gene identifiers and annotation components as the data will become accessible to other API users. Developers of online services may use the API to allow users to directly submit their sets for analysis.

## Discussion

### Similar tools

To our knowledge, no tool in Bioinformatics exists in isolation with the unique function of helping users build and manage sets of genes. Although the Gene Set Builder system shares similar properties with other multi-gene tools such as the Sequence Retrieval System (SRS) [13], SeqHound [14], and WebGestalt [15], it does not share the same fundamental concept, nor does it fit into the same categorical niche. Gene Set Builder's primary role is to help users build, annotate, and import gene sets in detail. SRS and SeqHound are focused more towards the computational aspects of working with a set of genes, not the long-term management and sharing of sets. WebGestalt provides users with an array of set analysis functions, but it does not facilitate the creation, maintenance, and sharing of sets. We are exploring mechanisms to directly submit Gene Set Builder sets to other tools such as WebGestalt.

### Benefits

The utility of Gene Set Builder offers users three major benefits: (1) it can help users annotate a pre-existing set of genes through synchronization with the Ensembl database to obtain alternate identifiers and descriptions; (2) it can aide in collaborative efforts by allowing team members to store gene sets in a central location where they can be easily accessed; and (3) it can be used as an aide for publication by allowing users to share their sets of genes with the community at large.

Most importantly, Gene Set Builder facilitates the storage of gene sets in a relational database as opposed to a text file, while offering a friendly environment that automates time-consuming tasks. The application's searching, annotating, and sharing features give users flexibility and convenience. Thus, users benefit from access to curated sets provided by other users, from the capacity to build sets in collaboration with others, and from the ability to shift from one set of identifiers to another.

### Limitations

While Gene Set Builder offers advantages to users, it does have several technical limitations which we hope to address in the future. One limitation involves Gene Set Builder's reliance upon Ensembl and GeneLynx for annotation data. Due to this dependency, users cannot build sets of prokaryotic or viral genes, nor include genes from non-supported eukaryotic organisms. Ideally, Gene Set Builder would eventually interact with systems such as Entrez Gene, the UCSC Genome Browser [16], and the Comprehensive Microbial Resource at TIGR [17]. As similar API resources emerge or mature for these systems, we will work to expand Gene Set Builder's compatibility.

While Gene Set Builder is fully compatible with recent versions of the Internet Explorer and Firefox web browsers, it renders inconsistently when viewed in older releases of Netscape and Safari. These problems stem from insufficient JavaScript support. To overcome this difficulty, we have implemented a "safe mode" state which can be toggled to increase usability when the system is being accessed via a less compatible browser.

## Conclusion

To our knowledge, the creation of general purpose gene set building tools has remained virtually unexplored. Gene Set Builder is our vision of what an application of this type can provide. It fulfils the needs of users interested in forming, annotating, sharing and exporting sets of genes.

## Availability and requirements

The Gene Set Builder can be accessed via , and it is available without charge to all users. A guest account is available for those who are interested in testing the system. We recommended a monitor screen resolution of at least 800 by 600 pixels or greater (1024 by 768 pixels is preferred), in thousands of colours, and a recent web browser with JavaScript and the Macromedia Flash 7 plug-in installed and enabled.

## List of abbreviations used

API: Application Programming Interface; CSS: Cascading Style Sheets; DBI: Database interface; FASTA: Fast-All; GSB: Gene Set Builder; HTML: Hypertext Markup Language; UCSC: University of California at Santa Cruz; UI: User Interface.

## Authors' contributions

WWW conceptualized the idea and directed the development process. JSL programmed components and suggested approaches. DY designed the user interface, developed the software, and wrote the manuscript with revisions provided by JSL and WWW.

## Supplementary Material

Additional File 1 In the internal portion, gene and set objects are unified by the "Genes in sets" table, which use multiple key entries to assign genes into sets. This database structure allows gene and set objects to exist independently. The open-access tables, "API Genes" and "API Sets", are set up specifically for API connectivity. Sets of genes exported by the user for API use are copied into these two tables. Click here for file
