# Supplementary figures and images for: The Gene Set Builder: collation, curation, and distribution of sets of genes
Source: BMC Bioinformatics. 2005 Dec 21;6:305. doi: 10.1186/1471-2105-6-305 (PMC1351202; doi:10.1186/1471-2105-6-305)

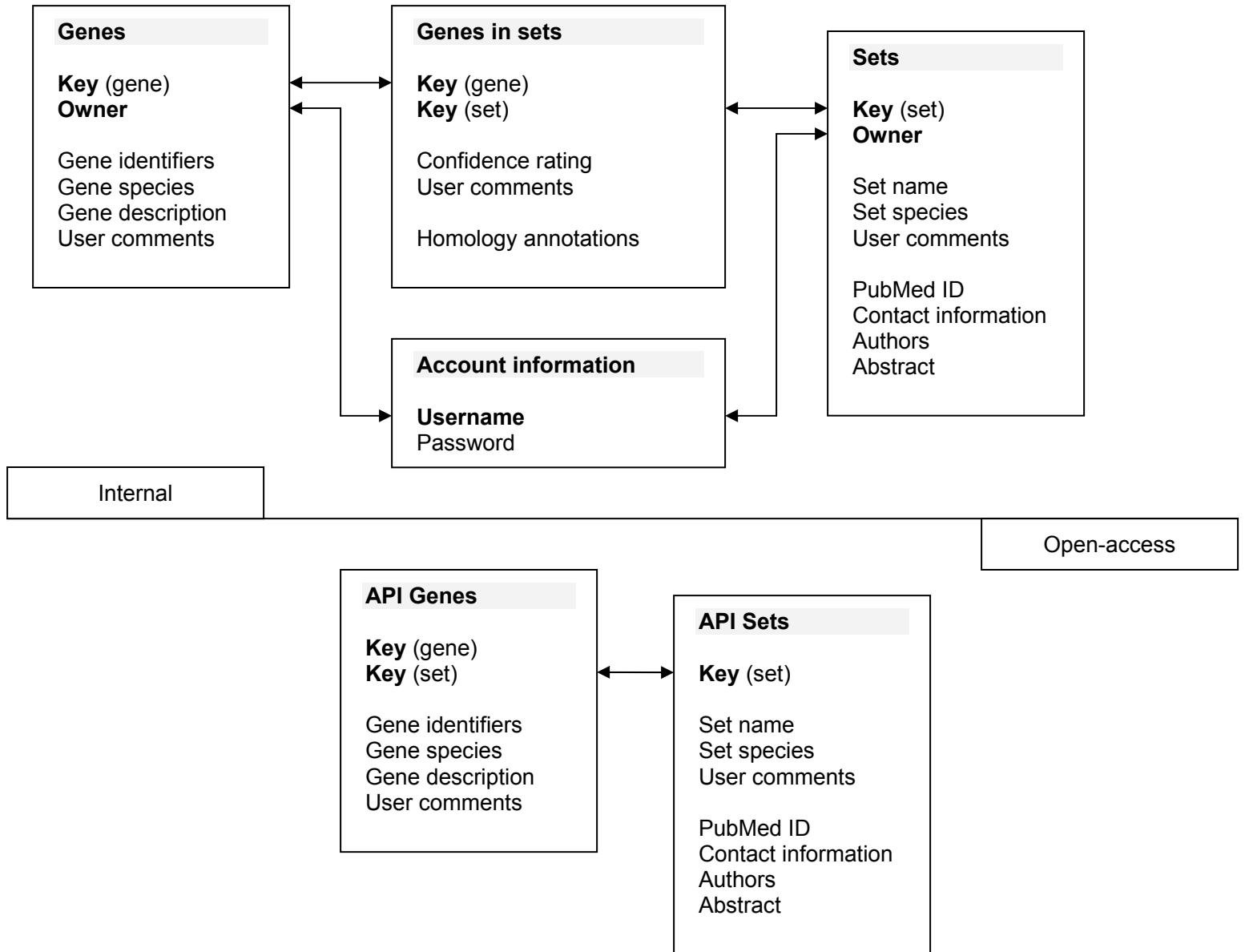

Supplement: Additional File 1 — In the internal portion, gene and set objects are unified by the "Genes in sets" table, which use multiple key entries to assign genes into sets. This database structure allows gene and set objects to exist independently. The open-access tables, "API Genes" and "API Sets", are set up specifically for API connectivity. Sets of genes exported by the user for API use are copied into these two tables. [file 1471-2105-6-305-S1.pdf]
